# Nontarget and Out-of-Field Doses from Electron Beam Radiotherapy

**DOI:** 10.3390/life12060858

**Published:** 2022-06-08

**Authors:** Natalia Matuszak, Marta Kruszyna-Mochalska, Agnieszka Skrobala, Adam Ryczkowski, Piotr Romanski, Igor Piotrowski, Katarzyna Kulcenty, Wiktoria Maria Suchorska, Julian Malicki

**Affiliations:** 1 Department of Electroradiology, Poznan University of Medical Sciences, 61-866 Poznan, Poland; marta.kruszyna@wco.pl (M.K.-M.); agnieszka.skrobala@wco.pl (A.S.); igor.piotrowski@wco.pl (I.P.); wiktoria.suchorska@wco.pl (W.M.S.); julian.malicki@wco.pl (J.M.); 2 Radiobiology Laboratory, Department of Medical Physics, Greater Poland Cancer Centre, 61-866 Poznan, Poland; katarzyna.kulcenty@wco.pl; 3 Department of Medical Physics, Greater Poland Cancer Centre, 61-866 Poznan, Poland; adam.ryczkowski@wco.pl (A.R.); piotr.romanski@wco.pl (P.R.)

**Keywords:** nontarget doses, out-of-field doses, electron beams, radiation scattering

## Abstract

In clinical radiotherapy, the most important aspects are the dose distribution in the target volume and healthy organs, including out-of-field doses in the body. Compared to photon beam radiation, dose distribution in electron beam radiotherapy has received much less attention, mainly due to the limited range of electrons in tissues. However, given the growing use of electron intraoperative radiotherapy and FLASH, further study is needed. Therefore, in this study, we determined out-of-field doses from an electron beam in a phantom model using two dosimetric detectors (diode E and cylindrical Farmer-type ionizing chamber) for electron energies of 6 MeV, 9 MeV and 12 MeV. We found a clear decrease in out-of-field doses as the distance from the field edge and depth increased. The out-of-field doses measured with the diode E were lower than those measured with the Farmer-type ionization chamber at each depth and for each electron energy level. The out-of-field doses increased when higher energy megavoltage electron beams were used (except for 9 MeV). The out-of-field doses at shallow depths (1 or 2 cm) declined rapidly up to a distance of 3 cm from the field edge. This study provides valuable data on the deposition of radiation energy from electron beams outside the irradiation field.

## 1. Introduction

Radiation scatter is an important concern in clinical dosimetry due to the risks it poses to the patient, mainly the potential to induce second cancers [1,2]. Radiation, either primary or Compton electrons, together with secondary photons (bremsstrahlung) from equipment parts or inside the body, are the main source of out-of-field radiation [3,4,5]. In both slab phantoms and human bodies, the scattered dose is due to radiation produced by the accelerator head and/or applicator, or to the interaction between primary and secondary radiation and the medium [6]. In both electron and photon beam radiotherapy, the patient’s body also produces scattered particles [7,8,9]. Unfortunately, to date no effective method has been identified to fully eliminate scattering. Similarly, despite attempts to design linear accelerators and applicators to eliminate radiation leakage, some leakage continues to occur.

Electron beams produced by megavoltage linear medical accelerators (up to 25 MeV) can only penetrate the body to shallow depths, which is why electron radiotherapy is limited to cancers located at or near the surface. The limited penetrating capacity of electrons also explains why research into scattered radiation from electron beams has received scant attention, especially compared to the vast body of literature on radiation scatter from photon beams [10,11,12,13,14,15]. Although electron beam radiotherapy is known to produce scattered electrons, out-of-field doses from this type of radiotherapy have not been well-characterised. Although the role of electron beams for external beam radiotherapy continues to decline, intraoperative electron radiotherapy (IOERT) is a commonly used technique, particularly for breast cancer [16]. Breast tissue is sensitive to radiation and IOERT in certain clinical situations, such as when the target volume is located near the heart, requires special consideration. Interest in FLASH radiotherapy with electron beams is growing due to its potential for normal tissues sparing [16,17]. These two modalities—IOERT and FLASH—both of which use electron beams, provide substantial rationale to perform studies in order to better characterize out-of-field doses from megavoltage electron beams. However, precise dosimetric measurement of out-of-field doses is challenging due to the limited knowledge of the physical characteristic of scattered radiation caused by electron megavoltage beams, and insufficient data on the response of detectors used to measure doses from these types of beams [18].

The main aim of the study was to measure out-of-field doses from electron beams in a slab phantom and to determine their dependence on depth. We investigated out-of-field doses for 6 MeV, 9 MeV, and 12 MeV electrons, which are the most commonly used energies or clinical radiotherapy, including IOERT. Higher electron energies (up to 25 MeV) are rarely used for radiotherapy. Additionally, we assessed the value of two different types of dosimetric detectors—the diode E and a cylindrical Farmer-type ionizing chamber—to measure out-of-field doses.

## 2. Materials and Methods

**Figure 1:**
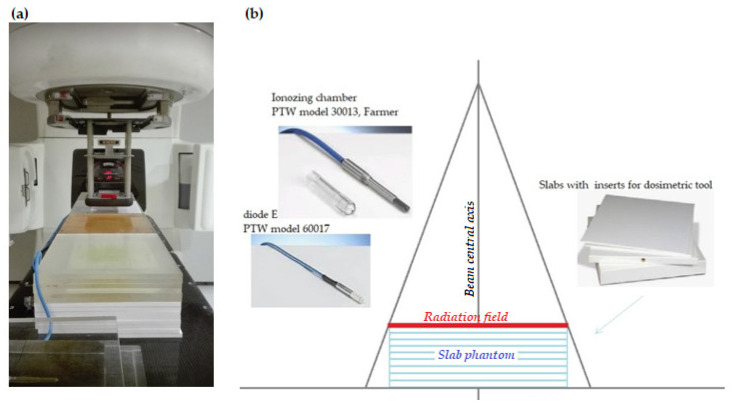
The solid state, purpose-built slab phantom: (**a**) photograph of the set-up, (**b**) graphic presentation.

This study was performed in a solid state, purpose-built slab phantom (Figure 1). The phantom was irradiated with a Varian Clinac 2300 linear accelerator (Varian, Palo Alto, CA, USA) at three different electron energy levels: 6 MeV, 9 MeV, and 12 MeV.

**Figure 2:**
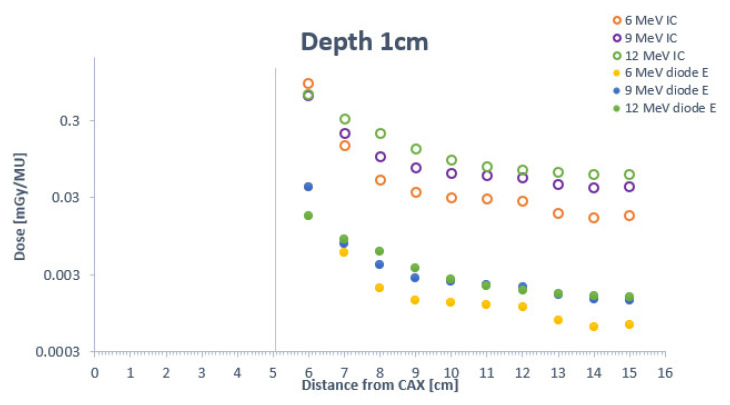

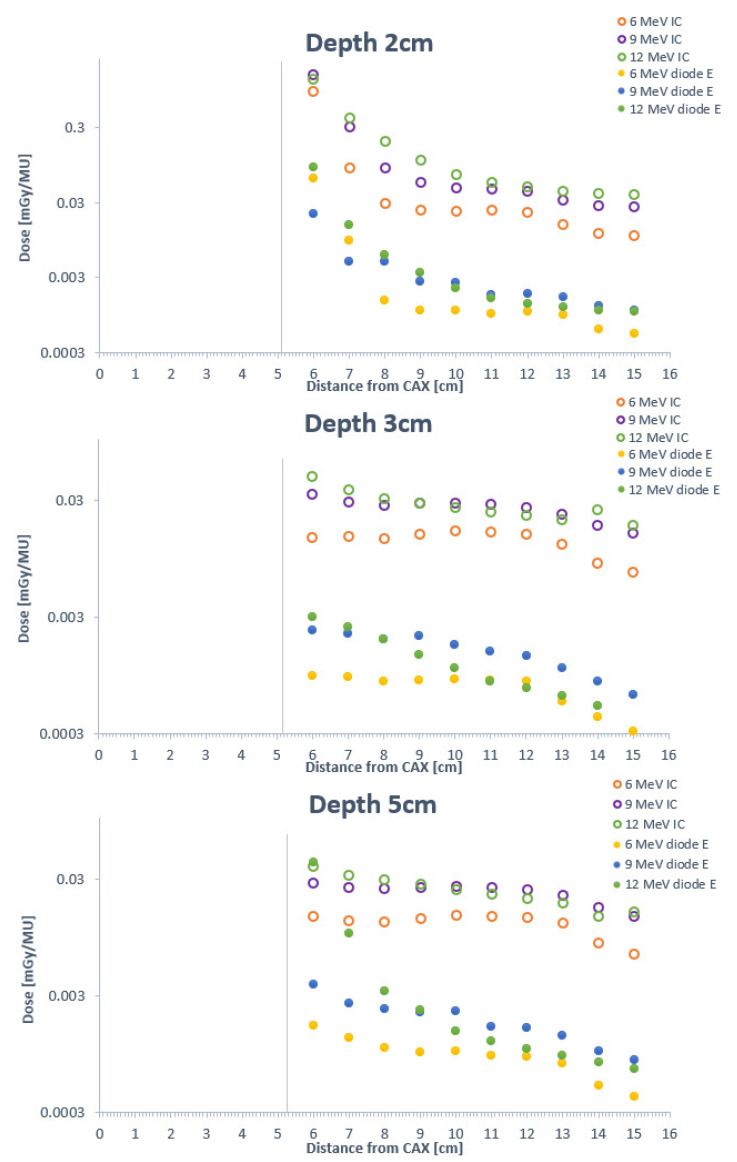
Comparison of out-of-field electron doses for 6 MeV, 9 MeV, 12 MeV beam energies measured by the cylindrical ionizing chamber and the diode E at depths of 1, 2, 3, and 5 cm at distances ranging from 1–10 cm. The vertical line corresponds to the edge of the radiation field at the given depth.

**Figure 3:**
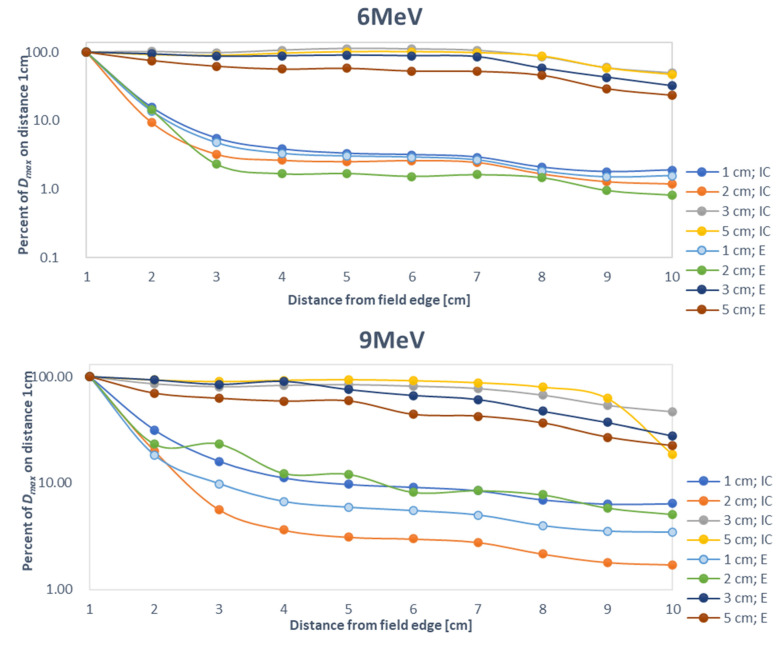

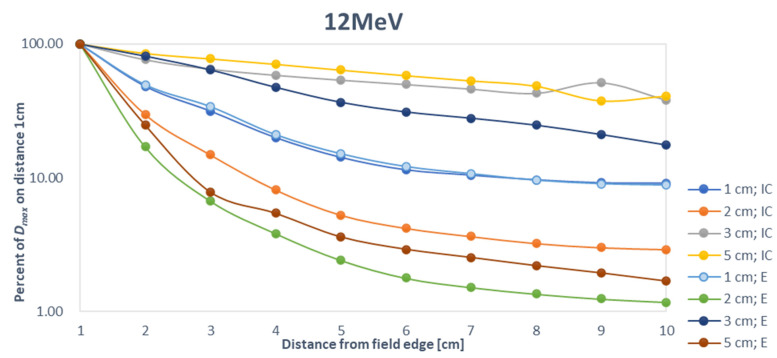
Comparison of out-of-field doses measured with a cylindrical ionizing chamber and diode E at depths of 1, 2, 3, and 5 cm and at distances ranging from 1–10 cm at different electron beam energies (6 MeV, 9 MeV, 12 MeV).

Out-of-field doses were measured at a range of different depths and distances from the beam central axis (CAX), as follows: (1) in increments of 1 cm (from 1 to 10 cm) from the field edge and (2) at depths of 1, 2, 3, and 5 cm from the surface. The doses’ values were recalculated to 1 MU for presentation in Figure 2 and normalised at 1 cm outside the edge of the field for presentation in Figure 3. 

All measurements were performed using two different types of dosimetric detectors (both capable of measuring doses from electron beams) to ensure more accurate readings, as follows: (1) a fully guarded cylindrical ionizing chamber (PTW model 30013, Farmer) and (2) the diode E (PTW model 60017). Both detectors are waterproof and suitable for use in solid state phantoms. The Farmer-type ionizing chamber is the standard ionization chamber for absolute dosimetry in high-energy photon, electron and proton beams. It has a vented cylindrical-shaped air volume (nominal volume, 0.6 cm^3^). Due to the relatively low voltage, there is no avalanche effect and no dead time [19]. However, dose readings are dependent on the radiation energy, particularly for low energy particles from scattered radiation, and the fact that calibration was done at the energy of the megavoltage beam had to be taken into account [19].

The waterproof silicon Diode E (nominal volume, 0.03 mm^3^) was designed for dosimetry in megavoltage electron and photon beams. However, it is suitable for dose measurements in small electron and photon fields, as well as for IOERT and dynamic and stereotactic techniques. Moreover, this detector shows excellent spatial resolution, with a response that is highly independent of the radiation energy. This is important given that the out-of-field energy from scattered radiation in a phantom is ten to few-hundred times lower than in the primary beam [20]. 

The nominal response for the cylindrical Farmer ionizing chamber is 20 nC/Gy and 9 nC/Gy for the diode E. The thin entrance window of the diode E enables measurements near the surface. The Farmer-type ionizing chamber was included for comparison because it is the most commonly used dosimetric tool.

## 3. Results

The figures below summarize the main findings. In general, we found that the out-of-field dose for electron beam energy decreased as both depth and distance increased beyond the field edge. The largest dose decrease (2–5% of the CAX dose) was observed from a 6 MeV beam at 3 cm from the field edge at depths of 1 and 2 cm as measured by the ionizing chamber and the diode E. At greater depths, the differences in out-of-field doses decreased the closer they were to the field edge. The smallest differences between the doses measured at 3 and 5 cm in depth were observed for the 12 MeV electron beam at a distance of 3 cm measured with the ionization chamber (difference = 3.2%) and for the 6 MeV electron beam energy at a distance of 6 cm measured with the diode E (difference = 6%).

Figure 2 illustrates how higher energy levels are associated with greater out-of-field doses. Out-of-field doses measured with the ionizing chamber decreased at depths of 1 cm and 2 cm. However, these doses increased when higher energy megavoltage electron beams were used, except for 9 MeV. There was a plateau from 3–7 cm from the field edge, which is clearly confirmed by the ionizing chamber measurements. In terms of the dose distribution at depths of 3 and 5 cm, the curves for both the 6 MeV and 9 MeV beams indicate a slight increase in out-of-field doses. Doses from the 9 MeV beam are larger than those originating from the 12 MeV beam. At distances > 7 cm and >9 cm, the dose decline is similar for both the 6 and 9 MeV energy beams.

The out-of-field doses at shallow depths (1 or 2 cm) decline rapidly up to a distance of 3 cm from the field edge and then plateau (in most cases) from 3–7 cm from the edge. At depths of 3 and 5 cm, the doses are homogeneous at all points along the path from 1–7 cm. For 12 MeV energies, the measurement obtained with the ionization chamber and diode E coincided in showing that out-of-field doses decrease as the depth increases.

Lower doses did not always correspond with shallower depths. The doses recorded at a depth of 3 cm were only 8% of those observed at 5 cm. As our results show, the depth-dose curves were not continuous downward sloping curves as a function of increasing distance from the field edge. For example, at 2 and 3 cm of depth, the curve reveals higher out-of-field doses and steeper penumbra than the dose distribution at 1 cm. For distances > 3 cm at a depth of 2 cm, the curve shows lower doses towards the 1 cm depth level. From this distance, both curves show a soft gradient, with a slight decrease in out-of-field doses. As expected, the dose decreased as a function of increasing distance from the field edge, with a plateau 4–8 cm from the edge. The increase in electron beam energy corresponded with a greater differential in out-of-field doses at the various depths, resulting in a more softly sloped dose curve. The largest variation in out-of-field doses between the various electron beam energies was observed between the 6 and 12 MeV beams (measured with the diode E): at 1 cm from the field edge (6 MeV beam), the dose at a depth of 5 cm was <5% of the dose at 1 cm; by contrast, the dose at 5 cm in depth for 12 MeV beams was <50% of the dose at a depth of 1 cm.

Table 1 and Figure 3 show the measurements at various depths, indicating detector dependence on the distance from field edge. Table 1 shows the doses (see limitations in the Section 4). The results are compared according to detector type. The out-of-field doses measured with the diode E were lower than those measured with the Farmer-type chamber at each depth level and for each energy beam level. Moreover, doses at 3 cm of depth were lower than those at 5 cm. Table 1 shows the percentage of out-of-field doses measured by the ionizing chamber compared to the out-of-field doses measured by diode E.

As Table 1 shows, at distances of 1 and 10 cm, the variation in out-of-field doses registered at a depth of 5 cm was greater than the variation observed at 1 cm. A lower percentage of out-of-field doses indicates a greater disagreement between detector types for doses at a given distance and depth.

Figure 3 shows the registered doses after normalisation to a dose value measured at the field edge. Normalisation helps to highlight the gradient along first few centimetres from field edge as a function of depth and shows the deviation between measurement results for the two types of detectors. The figure shows the out-of-field doses at distances ranging from 2–10 cm from the field edge, given as a percentage of the dose at the edge. For 6 and 9 MeV energy beams, minimal deviations between detectors can be observed at depths of 3 and 5 cm, suggesting that dose variability at 10 cm from the treatment field is negligible. For 12 MeV energy beams, the slope of the out-of-field doses was pronounced at each depth. Increasing the beam energy leads to a more stable fall-off of out-of-field doses as a function of out-of-field distance.

## 4. Discussion

During irradiation, out-of-field doses to various nontarget body parts are unavoidable due to the inherent properties of radiation. However, doses to healthy tissues should be kept as low as possible while ensuring that the curative effect is not compromised. The factors influencing changes in the treatment plan (beam configuration and dose constraints) are associated with: (a) advances in technology, which has led to new, more accurate therapeutic and dosimetric equipment, (b) advances in our understanding of the interaction between radiation and the medium, and (c) the growing body of knowledge on how cells, tissues, organs and the whole organism react to peripheral doses after radiotherapy.

Compared to conventional external beam radiotherapy, IOERT and FLASH techniques are based on a different concept of tumour irradiation, which is essential to characterize the out-of-field doses caused by these techniques.

In the literature, the term “nontarget” has a clinical connotation as it refers to a region outside the target. Technically, during multi-beam radiotherapy, the nontarget region receives doses from both the primary beam and scattered radiation. If only one beam is used (as occurs in IOERT and FLASH), the term nontarget refers to out-of-field regions. In the present study, we determined doses in a slab phantom outside the primary beam for single beam techniques with three different energy levels (6 MeV, 9 MeV, 12 MeV), which is why we consistently use the term “out-of-field” rather than “nontarget”. We also assessed the dependence of the dose on depth and distance from the CAX. We evaluated two different types of dosimetric detectors (diode E and Farmer-type ionizing chamber).

As expected, our results show that out-of-field doses generally decrease as a function of distance from the field edge. We found that the decrease in the radiation dose slows or remains constant as the distance from the CAX increases, consistent with the findings described by Cardenas et al. [21], potentially due to the increased obliquity angle of the electrons with off-axis distances. At depths of 3 and 5 cm, the out-of-field electron doses were relatively uniform, confirming dose deposition by primary electrons across the first few (1–2) centimetres such that the remaining dose at greater depths (≥3 cm) is merely residual of radiation bremsstrahlung. Finally, we also showed that the Farmer-type chamber—which is more commonly used to measure photon beam energy—can be utilized to verify the data obtained with the diode E in order to accurately determine electron dosimetry.

We found that when larger electron beam energies were used, the out-of-field doses were higher at greater depths, likely due to the characteristics of bremsstrahlung radiation, as described by Kry et al. [22] and Alabdoaburas et al. [9]. Interestingly, higher doses did not always correspond with shallower depths. We observed higher doses at greater depths due to the increase in electron fluency, the presence of secondary electrons, and electron scattering caused by scattering foils [23], the monitor chamber, and/or the electron applicator. Scattered electrons and bremsstrahlung photon contamination contribute to out-of-field doses, significantly so in the accelerator head and irradiated human tissue [7,24]. The probability of photon bremsstrahlung is proportional to the square of the atomic number of the material that the electrons impinge upon [25]. The backscattered electrons originating from the electron applicator are not deposited in the monitor chamber [26]. In weak shielded, out-of-field areas, the dose increase is due to escaping electrons. This finding is consistent with the findings of Alabdoaburas et al. [9], which suggests that the local peak dose is due to additional electron deposition [7]. Our results are partly consistent with the findings of Haghparast et al. [27], who found that peaks decreased as the depth increased. The curves over the entire distances from the field edge seem more homogeneous with increasing depth, in contrast to the reduction in explicit peaks. Uniformity of out-of-field doses with increasing depth may be due to the low penetration depth of electrons, which leads to a lower amount of scattered radiation at higher depths.

Given that electron applicators give off scattered radiation, Hogstrom et al. [28] proposed a multipurpose, retractable, accessory-less accelerator, which would be more advantageous in terms of delivery of mixed-beam therapy and intensity-modulated electron therapy. As Lax and Brahme [29] reported, the source of out-of-field electron scatter is the collimator edge, not the scattering foil. Those authors found that electron energy scattered from the collimator edge accounted for 40% of the hitting electron energy. The magnitude of scattered radiation is dependent on the beam energy level [30], which suggests that higher beam energy has a greater penetration capacity [12]. High out-of-field doses are more likely for high energy electron beams. Yeboah et al. [31] showed that out-of-field doses can reach around 5% of the CAX D_max_.

Our findings show a clear decrease in out-of-field doses as the distance from the field edge and depth increases. In addition, the main component of these out-of-field doses is scattered radiation, which is difficult to predict due to differences between linear accelerators in terms of construction and components. As a result, out-of-field doses may vary from study to study, leading to divergent results and making it difficult to reach definitive conclusions. That said, the correlation between out-of-field doses with distance, depth, and energy beam levels in our study was largely consistent with previous reports [9,21,27,31]. Crucially, higher beam energy leads to higher electron contamination [32]. Although out-of-field electron doses may appear to be less significant than those observed with photon beams, the deposition of radiation in sensitive volumes is evident and may cause unwanted damage to healthy tissues, just as occurs with photon beam energy.

### Strengths and Limitations

This study has several limitations. First, angular scattering in real irradiation (i.e., in patients) differ from those obtained in phantom models (due to irregularities in human bodies), which in turn affects out-of-field doses. Second, the available dosimetric tools are imperfect, and the phantom layout could also contribute to increased scattering. Third, we only assessed the nontarget doses from scattered radiation; we were unable to evaluate the effects of multi-beam techniques, in which primary electrons from one of the beams has an outsized contribution to elevating the nontarget dose. Although this situation is highly relevant clinically, it was outside the scope of our study due to the multiple physical mechanisms underlying the interactions and the challenges associated with dosimetric measurements. Fourth, dosimetric determination of doses from scattered radiation is challenging due to the difficulty in reliably determining a calibration factor. The detector response depends on the particle energy. To overcome this challenge, we applied correction factors as has been done in similar studies [33,34].

Finally, although some aspects of this study considered basic relationships between dosimetric parameters and out-of-field doses, we believe this was necessary to illustrate the large differences in out-of-field doses when using other detectors.

## 5. Conclusions

Our findings show a clear decrease in out-of-field doses as the distance from the field edge and depth increases. The out-of-field doses are caused by radiation scatter originating from the applicators of the linear accelerator and in the medium. However, it is difficult to predict these doses due to the many differences among linear accelerators in terms of the construction and components.

In this study, we have demonstrated that it is possible to measure out-of-field electron doses using a cylindrical Farmer-type ionization chamber, even though these tools are more commonly used for photon dosimetry. Further studies could consider using other detectors or other techniques, such as Monte Carlo simulation, with the aim of more accurately determining the out-of-field dose distribution.

## Figures and Tables

**Table 1:** Percent dose at a given depth and distance as (DE/DIC) *100%.

	6 MeV		9 MeV		12 MeV	
Depth	Distance from Field Edge					
	1 cm	10 cm	1 cm	10 cm	1 cm	10 cm
1 cm	4.5%	3.8%	6.3%	3.4%	2.7%	2.5%
5 cm	11.5%	6.0%	13.7%	5.5%	3.9%	4.5%

Abbreviations: DE indicates the dose measured by the diode E; DIC indicates the dose measured by the cylindrical Farmer-type ionizing chamber.

## Data Availability

The data that support the findings of this study are available from the corresponding author upon request.

## References

[1] Athiyaman H., Mayilvaganan A., Chougule A., Joan M., Kumar H.S. (2019). Estimation of radiation-induced second cancer risk associated with the institutional field matching craniospinal irradiation technique: A comparative treatment planning study. Rep. Pract. Oncol. Radiother..

[2] König L., Haering P., Lang C., Splinter M., von Nettelbladt B., Weykamp F., Hoegen P., Lischalk J.W., Herfarth K., Debus J. (2020). Secondary malignancy risk following proton vs. X-ray treatment of mediastinal malignant lymphoma: A comparative modeling study of thoracic organ-specific cancer risk. Front. Oncol..

[3] Podgoršak E.B., International Atomic Energy Agency (2005). Radiation Oncology Physics: A Handbook for Teachers and Students.

[4] Hogstrom K.R., Almond P.R. (2006). Review of electron beam therapy physics. Phys. Med. Biol..

[5] Kruszyna-Mochalska M., Skrobala A., Romanski P., Ryczkowski A., Suchorska W., Kulcenty K., Piotrowski I., Borowicz D., Graczyk K., Matuszak N. (2022). Influence of specific treatment parameters on nontarget and out-of-field doses in a phantom model of prostate SBRT with CyberKnife and TrueBeam. Life.

[6] Kruszyna M., Adamczyk S., Skrobała A., Skórska M., Suchorska W., Zaleska K., Kowalik A., Jackowiak W., Malicki J. (2017). Low dose out-of-field radiotherapy, part 1: Measurement of scattered doses. Cancer Radiothér..

[7] Zhu T.C., Das I.J., Bjarngard B.E. (2001). Characteristics of bremsstrahlung in electron beams. Med. Phys..

[8] Gerbi B.J., Antolak J.A., Deibel F.C., Followill D.S., Herman M.G., Higgins P.D., Huq M.S., Mihailidis D.N., Yorke E.D., Hogstrom K.R. (2009). Recommendations for clinical electron beam dosimetry: Supplement to the recommendations of Task Group 25. Med. Phys..

[9] Alabdoaburas M.M., Mege J.-P., Chavaudra J., Bezin J.V., Veres A., de Vathaire F., Lefkopoulos D., Diallo I. (2015). Experimental assessment of out-of-field dose components in high energy electron beams used in external beam radiotherapy. J. Appl. Clin. Med. Phys..

[10] Shimozato T., Okudaira K., Fuse H., Tabushi K. (2013). Monte Carlo simulation and measurement of radiation leakage from applicators used in external electron radiotherapy. Phys. Med..

[11] Kry S.F., Vassiliev O.N., Mohan R. (2010). Out-of-field photon dose following removal of the flattening filter from a medical accelerator. Phys. Med. Biol..

[12] Van Battum L.J., van der Zee W., Huizenga H. (2003). Scattered radiation from applicators in clinical electron beams. Phys. Med. Biol..

[13] Perec A., Kubo H. (1990). Radiation leakage through electron applicators on Clinac-1800 accelerators. Med. Phys..

[14] Bordy J.M., Bessieres I., D’Agostino E., Domingo C., D’Errico F., di Fulvio A., Knežević Ž., Miljanić S., Olko P., Ostrowsky A. (2013). Radiotherapy out-of-field dosimetry: Experimental and computational results for photons in a water tank. Radiat. Meas..

[15] Harrison R. (2017). Out-of-field doses in radiotherapy: Input to epidemiological studies and dose-risk models. Phys. Med..

[16] Sedlmayer F., Reitsamer R., Fussl C., Ziegler I., Zehentmayr F., Deutschmann H., Kopp P., Fastner G. (2014). Boost IORT in breast cancer: Body of evidence. Int. J. Breast Cancer.

[17] Favaudon V., Caplier L., Monceau V., Pouzoulet F., Sayarath M., Fouillade C., Poupon M.F., Brito I., Hupé P., Bourhis J. (2014). Ultrahigh dose-rate FLASH irradiation increases the differential response between normal and tumour tissue in mice. Sci. Transl. Med..

[18] Matuszak N., Suchorska W.M., Milecki P., Kruszyna-Mochalska M., Misiarz A., Pracz J., Malicki J. (2022). FLASH radiotherapy: An emerging approach in radiation therapy. Rep. Pract. Oncol. Radiother..

[19] Bayatiani M., Fallahi F., Aliasgharzadeh A., Ghorbani M., Khajetash B., Seif F. (2021). A comparison of symmetry and flatness measurements in small electron fields by different dosimeters in electron beam radiotherapy. Rep. Pract. Oncol. Radiother..

[20] Skrobala A., Adamczyk S., Kruszyna-Mochalska M., Skórska M., Konefał A., Suchorska W., Zaleska K., Kowalik A., Jackowiak W., Malicki J. (2017). Low dose out-of-field radiotherapy, part 2: Calculating the mean photon energy values for the out-of-field photon energy spectrum from scattered radiation using Monte Carlo methods. Cancer Radiothér..

[21] Cardenas C.E., Nitsch P.L., Kudchadker R.J., Howell R.M., Kry S.F. (2016). Out-of-field doses and neutron dose equivalents for electron beams from modern Varian and Elekta linear accelerators. J. Appl. Clin. Med. Phys..

[22] Kry S.F., Bednarz B., Howell R.M., Dauer L., Followill D., Klein E., Paganetti H., Wang B., Wuu C.-S., Xu X.G. (2017). AAPM TG 158: Measurement and calculation of doses outside the treated volume from external-beam radiation therapy. Med. Phys..

[23] Bieda M.R., Antolak J.A., Hogstrom K.R. (2001). The effect of scattering foil parameters on electron-beam Monte Carlo calculations. Med. Phys..

[24] Chow J.C., Grigorov G.N. (2006). Peripheral dose outside applicators in electron beams. Phys. Med. Biol..

[25] Jabbari N., Hashemi-Malayeri B., Farajollahi A.R., Kazemnejad A. (2007). Monte Carlo calculation of scattered radiation from applicators in low energy clinical electron beams. Nukleonika.

[26] Verhaegen F., Symonds-Tayler R., Liu H.H., Nahum A.E. (2000). Backscatter towards the monitor ion chamber in high-energy photon and electron beams: Charge integration versus Monte Carlo simulation. Phys. Med. Biol..

[27] Haghparast A., Amiri F., Yarahmadi M., Rezaei M. (2018). The peripheral dose outside the applicator in electron beams of an Elekta linear accelerator. Australas. Phys. Eng. Sci. Med..

[28] Hogstrom K.R., Perez C.A., Brady L.W., Halperin E.C., Schmidt-Ullrich R.K. (2004). Electron beam therapy: Dosimetry, planning, and techniques. Principles and Practice of Radiation Oncology.

[29] Lax I., Brahme A. (1980). Collimation of high energy electron beams. Acta Radiol. Oncol..

[30] Van Battum L.J., Huizenga H. (1999). On the initial angular variances of clinical electron beams. Phys. Med. Biol..

[31] Yeboah C., Karotki A., Hunt D., Holly R. (2010). Quantification and reduction of peripheral dose from leakage radiation on Siemens primus accelerators in electron therapy mode. J. Appl. Clin. Med. Phys..

[32] Yani S., Budiansah I., Rhani M.F., Haryanto F. (2020). Monte Carlo model and output factors of Elekta infinity™ 6 and 10 MV photon beam. Rep. Pract. Oncol. Radiother..

[33] Kaderka R., Schardt D., Durante M., Berger T., Ramm U., Licher J., La Tessa C. (2012). Out-of-field dose measurements in a water phantom using different radiotherapy modalities. Phys. Med. Biol..

[34] Yoon J., Heins D., Zhao X., Sanders M., Zhang R. (2017). Measurement and modeling of out-of-field doses from various advanced post-mastectomy radiotherapy techniques. Phys. Med. Biol..

